# Dietary Determinants of and Possible Solutions to Iron Deficiency for Young Women Living in Industrialized Countries: A Review

**DOI:** 10.3390/nu6093747

**Published:** 2014-09-19

**Authors:** Kathryn L. Beck, Cathryn A. Conlon, Rozanne Kruger, Jane Coad

**Affiliations:** Institute of Food Nutrition and Human Health, College of Health, Massey University, Private Bag 102 904, North Shore City 0745, New Zealand; E-Mails: c.conlon@massey.ac.nz (C.A.C.); r.kruger@massey.ac.nz (R.K.); j.coad@massey.ac.nz (J.C.)

**Keywords:** iron deficiency, anemia, diet and iron status, heme and non-heme iron, dietary patterns, inhibitors and enhancers of iron absorption, treatment of iron deficiency

## Abstract

Iron deficiency is a concern in both developing and developed (industrialized) countries; and young women are particularly vulnerable. This review investigates dietary determinants of and possible solutions to iron deficiency in young women living in industrialized countries. Dietary factors including ascorbic acid and an elusive factor in animal protein foods (meat; fish and poultry) enhance iron absorption; while phytic acid; soy protein; calcium and polyphenols inhibit iron absorption. However; the effects of these dietary factors on iron absorption do not necessarily translate into an association with iron status and iron stores (serum ferritin concentration). In cross-sectional studies; only meat intake has consistently (positively) been associated with higher serum ferritin concentrations. The enhancing effects of ascorbic acid and meat on iron absorption may be negated by the simultaneous consumption of foods and nutrients which are inhibitory. Recent cross-sectional studies have considered the combination and timing of foods consumed; with mixed results. Dietary interventions using a range of focused dietary measures to improve iron status appear to be more effective than dietary approaches that focus on single nutrients or foods. Further research is needed to determine optimal dietary recommendations for both the prevention and treatment of iron deficiency.

## 1. Introduction

Nearly one-quarter of the population worldwide are affected by anemia, of which iron deficiency is the primary cause [1,2]. Iron deficiency is associated with impaired physical work capacity [3], reduced mood and cognitive function [4], and poor pregnancy related outcomes [5,6]. An individual’s iron status falls on a continuum, ranging from replete iron stores, through to depleted iron stores, iron deficiency and iron deficiency anemia [7]. Individuals with iron deficiency are, therefore, at increased risk of developing iron deficiency anemia.

Despite advances in healthcare, iron deficiency remains a major public health concern in both industrialized and non-industrialized (developing) countries, with young women being particularly vulnerable. In industrialized countries, iron deficiency should be easily identified and treated, and yet it is often overlooked by medical practitioners [8] or not recognized by women as a concern. Therefore even in these countries innovative approaches to the prevention and treatment of iron deficiency are required [9].

Dietary factors play a role in the development of iron deficiency and subsequent development of iron deficiency anemia. This purpose of this review is to discuss the most recent evidence regarding dietary determinants and possible solutions to iron deficiency for young women living in industrialized countries. Dietary factors affecting iron absorption will be outlined, and cross-sectional studies reviewed to determine associations between dietary intake and iron status in young women. The efficacy of dietary interventions aimed at improving iron status in young women will be reviewed. In addition the iron status and iron intakes of women living in industrialized countries (United States of America (USA), Canada, Australia, New Zealand, and the United Kingdom (UK)) will be described, as well as the latest recommended intakes for iron for young women living in these countries. The effect of iron intake from supplements or fortified foods on iron status is reviewed elsewhere [10] and is outside the scope of this review article.

## 2. Methods

A search strategy was undertaken to identify reports and studies addressing the prevalence of iron deficiency, investigating dietary determinants of iron deficiency and the efficacy of dietary interventions to treat iron deficiency in young women living in industrialized countries. A comprehensive search of PubMed was undertaken between February and July 2014 for publications in English for the period 1966–2014. The following search terms were used to identify relevant articles: iron, iron status, iron deficiency, iron deficiency anemia, diet, heme iron, non-heme iron, dietary patterns, meat, ascorbic acid, phytic acid, soy protein, calcium and polyphenols. To obtain data on iron status, iron intakes and recommended dietary intakes, national health and/or nutrition surveys and government publications were identified. Publications focused on young women living in industrialized English speaking countries (the USA, Canada, UK, Australia and New Zealand where available).

## 3. Prevalence of Iron Deficiency in Young Women Living in Industrialized Countries

To be able to compare and contrast the prevalence of iron deficiency between countries, it is important to address the spectrum of iron deficiency. This is typically characterized by three phases: iron depletion, iron deficiency (iron deficient erythropoiesis or non-anemic iron deficiency), and iron deficiency anemia [7]. The terminology and cut-off values for the biochemical measures of each of these stages often vary. In the first phase (iron depletion), the body’s stores of iron decrease, reflected by a reduction in serum ferritin concentration [7]. During the second phase (iron deficiency), tissue iron stores also decrease, and some iron-dependent functions are compromised. Serum ferritin concentrations are further reduced, serum iron decreases and total iron-binding capacity increases, resulting in a fall in transferrin saturation [7]. In this phase, soluble transferrin receptor and zinc protoporphyrin concentrations increase. In the final phase (iron deficiency anemia), oxygen supply to the tissues becomes impaired, which is reflected by a decrease in hemoglobin concentrations [7].

For estimates of global iron deficiency, the prevalence of anemia is often used as a proxy indicator [11]. Although iron deficiency is the primary cause of anemia, other factors also contribute, thus, this approach needs to be used with caution. The prevalence of iron deficiency and iron deficiency anemia is higher in developing countries than in industrialized countries, due to factors, such as infections (hookworm and malaria), causing blood loss and diets with very low iron content and low bioavailability [12,13] However, prevalence rates of iron deficiency without anemia in industrialized countries ranges from 5.2% to 16.6% (Table 1).

## 4. Recommended Iron Intakes for Young Women Living in Industrialized 

Most industrialized countries have established recommended values for iron intake based on available scientific evidence. The estimated average requirement (EAR) is the amount of iron estimated to meet the needs of 50% of individuals in a population. From the EAR, the recommendations for adequacy of individual daily iron intakes can be derived. The recommended dietary allowance (RDA) is the amount of iron that meets the requirements of 97.5% of a population. Average intakes (AI) are used when RDA cannot be determined, while the Tolerable Upper Intake Level guides against excessive intakes which may adversely affect health [14]. Most industrialized countries follow a similar approach, although terminology may vary, for example, in the USA and Canada, the RDA is used, in Australia and New Zealand, the Recommended Dietary Intake (RDI), and, in the UK, Recommended Nutrient Intake (RNI).

**Table 1:** National prevalence data for iron deficiency and iron deficiency anemia for young women from industrialized countries from national nutrition and health surveys ^a^.

Country	Participants	Iron Status of Participants
Canada	2009–2011 Canadian Health Measures Survey [15] 2042 females, 20–49 y	90.9%—SF sufficiency (SF ≥ 15 μg/L) 96.3%—Hb sufficiency (Hb ≥ 120 g/L) Mean SF—41 μg/L/Mean Hb—135 g/L
New Zealand	2008/09 New Zealand National Adult Nutrition Survey [16] 240 females, 19–30 y	5.2%—ID (SF < 12 μg/L, zinc protoporphyrin > 60 μmol/mol) 1.2%—IDA (SF < 12 μg/L, zinc protoporphyrin > 60 μmol/mol, Hb < 120 g/L) Mean SF—52 μg/L / Mean Hb—134 g/L
New Zealand	2008/2009 New Zealand National Adult Nutrition Survey [16] 508 females, 31–50 y	12.1%—ID (SF < 12 μg/L, zinc protoporphyrin > 60 μmol/mol) 6.3%—IDA (SF < 12 μg/L, zinc protoporphyrin > 60 μmol/mol, Hb < 120 g/L) Mean SF—59 μg/L/Mean Hb—131 g/L
United Kingdom	September 2008–November 2010 UK National Diet and Nutrition Survey [17] Females, 19–64 y	16.6%—ID (SF < 15 μg/L) 3.3%—IDA (Hb < 120 g/L, SF < 15 μg/L) Mean SF—56 μg/L / Mean Hb—133 g/L
United States	NHANES 2003–2006 Females, 20–49 y [18]	13.2%—ID (SF < 15 μg/L) (20–49 year, *n* = 2539) Mean SF—38.1 μg/L (20–39 year, *n* = 1780)

^a^ Data are shown from countries where national prevalence data are available (no data available for Australia). Abbreviations: Hb—Hemoglobin; ID—Iron deficiency; IDA—Iron deficiency anemia; *n*—number; NHANES—National Health and Examination Survey; SF—Serum ferritin; UK—United Kingdom.

Iron recommendations are difficult to establish for several reasons, including difficulties in assessing iron intakes and the amount of iron needed to maintain iron stores. Blood (and subsequent iron) losses from menstruation are variable between women and difficult to measure [19]. The wide range of iron bioavailability of individual foods consumed within a diet also makes it difficult to estimate iron requirements accurately [20]. Iron bioavailability from a typical diet consumed by iron replete women is used to estimate iron recommendations; which may not accurately reflect requirements of all young women, particularly if they are within the spectrum of iron deficiency [20]. Recommendations for iron are estimated conservatively, and given that the RDI for iron is aimed to meet the requirements of nearly all of the population, it is likely that recommended intakes are in excess of actual requirements for many women [20]. It has recently been suggested that dietary recommendations for iron be based on a range of iron bioavailability factors (e.g., consumption of meat) and characteristics of the individual such as iron status [21].

Individuals in most industrialized countries consume diets of high iron bioavailability (>2.1 mg or >15% of iron absorbed daily) [22]. Diets of high iron bioavailability contain generous amounts of meat and foods that enhance iron absorption (e.g., contain ascorbic acid) and low intakes of foods that inhibit iron absorption (e.g., phytic acid). Differences in assumptions regarding the efficacy of iron absorbed and utilized gives rise to different recommendations between countries (see Table 2). Four of the major industrialized countries identified in this review have the same iron recommendations for non-pregnant, non-lactating women aged 19 to 50 years, namely a RDI and RDA of 18 mg/day (New Zealand, Australia, USA and Canada) [23,24]. The RNI for women living in the UK is 14.8 mg/day [25] (Table 2). Recommendations by the FAO and WHO are based on lower levels of iron absorption, and are therefore somewhat higher with RNI being 19.6 mg and 29.4 mg/day, respectively [26]. Plant based diets (e.g., in developing countries and those of people following vegetarian diets) have reduced levels of iron bioavailability (no meat or increased phytic acid from plant foods), and individuals following these diets are likely to have higher iron requirements [23].

**Table 2:** Recommendations for iron intake for non-pregnant, non-lactating women aged 19 to 50 years [23,24,25,26].

Females (Age, Years)	NZ and Australia ^a ^(mg)		UK ^b^ (mg)		USA and Canada ^a^ (mg)		FAO/WHO ^c^ (mg)	
	RDI ^d^	EAR ^e^	RNI ^d^	EAR ^e^	RDA ^d^	EAR ^e^	RNI ^d^	RNI ^d^
19–50	18.0	8.0	14.8	11.4	18.0	8.1		
18+							19.6	29.4

^a^ Based on 18% absorption; ^b^ Based on 15% absorption; ^c^ Based on 15% and 10% absorption, respectively; ^d^ Meets the needs of 97.5% of individuals in the age grouping; ^e^ Meets the needs of 50% of individuals in the age grouping. Abbreviations: RDA—Recommended Dietary Allowance; RDI—Recommended Dietary Intake; RNI—Reference Nutrient Intake; EAR—Estimated Average Requirement.

## 5. Current Dietary Intakes of Iron for Young Women Living in Industrialized Countries

Median and mean intakes of iron for young women living in industrialized countries range from 8.8 to 13.9 mg/day (Table 3). A small proportion does not meet the EAR for iron. For example, the estimated prevalence of inadequate intake of iron in New Zealand (determined using full probability analysis) was 6.0% for females aged 19 to 30 years and 15.4% for females aged 31 to 50 years [16].

## 6. Dietary and Non-Dietary Factors Regulating Iron Absorption

Iron balance is controlled by iron absorption by the gut enterocytes; there is no route of controlled excretion of iron. Iron absorption is regulated by dietary and systemic factors. Dietary iron is predominantly non-heme iron with about 5%–10% in the form of heme iron in diets containing meat [27]. Although heme iron constitutes a smaller part of dietary iron, it is highly bioavailable and 20%–30% of heme iron is absorbed. Absorption of non-heme iron is much more variable and significantly affected by other components of the diet, with 1%–10% of non-heme iron absorbed.

Iron in the environment and the diet is predominantly ferric iron (Fe^3+^) which is insoluble and so not bioavailable. Before it can be absorbed, non-heme iron has to be reduced from ferric (Fe^3+^) to ferrous (Fe^2+^) iron by dietary reducing agents, such as ascorbic acid or by endogenous ferri-reductases, such as duodenal cytochrome B (dcytB) [28]. Ferrous iron is transported across the apical membrane of the duodenum by the divalent metal transporter 1 (DMT1), which is localized on the brush border membrane close to dcytB. The uptake of ferrous ions by dcytB is driven by proton co-transport [29], so an acidic duodenal pH facilitates iron uptake, and is competitively inhibited by other divalent cations.

Ascorbic acid is the most effective enhancer of non-heme iron absorption. Other dietary factors including citric acid and other organic acids, alcohol and carotenes similarly enhance non-heme iron absorption [27]. Animal proteins, such as meat, fish, and poultry, enhance iron absorption but the bioactive component of the “meat factor” has yet to be identified. Meat also promotes non-heme iron absorption by stimulating gastric acid production. Absorption of non-heme iron is inhibited by phytic acid (inositol hexaphosphate and inositol pentaphosphate) in grains and cereals and by polyphenols in some vegetables, coffee, tea, and wine. These inhibitors chelate non-heme iron so it is not available for uptake.

Heme from the proteolytic digestion of dietary myoglobin and hemoglobin is transported as the intact porphyrin into the enterocyte by a less well characterized pathway, possibly a heme transport carrier [30], which may be inhibited by the presence of calcium. Once in the enterocyte, iron is released from heme by heme oygenase. Prolonged cooking of meat results in heme iron being converted to non-heme iron as the porphyrin ring is damaged [31]. It is suggested that dietary iron is also taken up as ferritin, the ubiquitous and highly conserved iron storage molecule of plants and animal cells. Ferritin is resistant to proteolytic digestion and probably interacts with a high affinity receptor before being taken into the enterocyte by endocytosis [32].

**Table 3:** Adequacy of population iron intakes among women living in industrialized countries.

Country	Survey	Participants	Dietary (Food) Iron Intakes (mg)	EAR (mg/Day)	RDI/RDA/RNI (mg/Day)	Inadequate Intakes (<EAR %)
Australia	1995 National Nutrition Survey [33]	19–24 y 25–44 y	10.6 ^a^ 11.1	8.0	18.0	
New Zealand	2008/2009 New Zealand National Adult Nutrition Survey [16]	19–30 y 31–50 y	10.2 (9.3–11.1) ^b^ 10.2 (9.6–10.8)	8.0	18.0	6.0 15.4
USA	NHANES 2001–2002 [34]	19–30 y (*n* = 465) 31–50 y (*n* = 754)	13.9 (0.56) ^c^ 13.4 ^a^ 13.1 (0.40) ^c^ 12.4 ^a^	8.1	18.0	15.0 17.0
USA	NHANES 2009–2010 [35]	20–29 y 30–39 y 40–49 y	13.5 (0.33) ^c^ 14.1 (0.38) 12.9 (0.49)	8.1	18.0	n/a
Canada	2004 Community Health Survey [36]	19–30 y (*n* = 1854) 31–50 y (*n* = 2686)	12.4 (0.3) ^c^ 12.1 (0.3) ^d^ 12.4 (0.2) ^c^ 12.1 (0.2) ^d^	7.7	18.0	16.8 (1.5) ^e^ 18.3 (1.1)
UK	September 2008/09–November 2010/11 UK National Diet and Nutrition Survey [37]	19–64 y	10.1 (4.0, 25.3) ^f^	11.4	14.8	20.0

^a^ Median; ^b^ Median (25%–75% IQR); ^c^ Mean (SE); ^d^ 50th Percentile (SE); ^e^ % inadequacy (SE); ^f^ Median (upper 2.5 percentile, lower 2.5 percentile). Abbreviations: RDA–—Recommended Dietary Allowance; RDI—Recommended Dietary Intake; RNI—Reference Nutrient Intake; EAR—Estimated Average Requirement; NHANES—National Health and Examination Survey.

Iron from all dietary sources enters a common intracellular pool from which it is either stored as ferritin in the enterocyte or exported from the enterocyte via the ferroportin transporter on the basal side of the cell. Ferroportin transports ferrous iron which is immediately oxidized to Fe^3+^ and picked up by transferrin to be transported to cells expressing transferrin receptors.

Iron absorption is affected by systemic factors. In iron deficiency, there is up-regulation of expression of DMT1 and ferroportin in new enterocytes differentiating from the stem cells in the villus crypts [38]. These cells are renewed every four to five days, which allows the uptake of iron to be sensitively controlled in response to need. If the gut is exposed to a high level of iron, DMT1 transporters are trafficked into the interior of the cell so non-heme iron absorption is reduced. In iron sufficiency, the expression of DMT1 and ferroportin is reduced. Hepcidin secretion by the liver increases in response to iron sufficiency; hepcidin binds to ferroportin causing it to be internalized. This not only reduces egress of iron from the enterocyte but also causes the sequestration of iron within the macrophages. The macrophages recycle iron from senescent red blood cells, contributing about 90%–95% of the daily iron requirements for erythropoiesis. Hepcidin also inhibits expression of DMT1 and dcytB on the apical membrane [39] so it abrogates iron uptake both into, as well as out of, the enterocyte. Hepcidin production is increased by infection and inflammatory conditions [40] thus limiting iron availability for pathogens [41]. This response is mediated by raised levels of pro-inflammatory cytokines and adipokines so chronic inflammatory conditions, including obesity, can result in iron being withheld from hemoglobin synthesis; the resulting iron deficiency anemia is refractory to dietary iron or oral supplements. Erythropoietin positively affects iron transport across the gut [27].

## 7. Dietary Determinants of Iron Status in Young Women Living in Industrialized Countries

There are a number of dietary factors which are known to affect iron absorption and, yet, studies have shown that these inhibiting and enhancing effects do not always impact on iron status. There are a number of reasons for this. Several studies have shown iron absorption measured over several meals or days to be less than that of iron absorption measured from single meals [42,43,44]. The enhancing effects of nutrients such as ascorbic acid on iron absorption may be negated by the simultaneous consumption of nutrients or foods which inhibit iron absorption. Finally, there are limitations in the designs of studies which have investigated associations or the effects of dietary interventions on iron status. These limitations are discussed in Section 7.11 and Section 8.5. As far as we are aware, no longitudinal studies have investigated associations between dietary intake and iron status in young women living in industrialized countries. A number of cross-sectional studies have been undertaken in industrialized countries to investigate associations between dietary intake and iron status in young women. The results of these studies are discussed below.

### 7.1. Dietary Iron

The majority of cross-sectional studies in young women have not found an association between total dietary iron intake and iron status [45,46,47,48,49,50,51,52,53,54,55,56,57,58,59]. Those studies showing a positive relationship included women aged 35 to 60 years [60], and a study which showed a positive relationship between iron intake and serum ferritin concentrations in smokers only [61]. The type of iron (heme* versus* non-heme) appears to be a more important determinant of iron status than total dietary intake [62].

### 7.2. Meat, Fish and Poultry/Heme Iron

Most studies in young women have observed a positive association between iron status and meat [45,48,54,57,59,60,63,64,65,66] or heme iron intake [49,53,54,60,67]. Only a few studies have found no association between meat or heme iron intake and iron status [50,55,61]. These have included a study in Japanese women [50], a study in 80 university students from the United States [61], and data from women in the UK National Diet and Nutrition Survey [55].

Other studies have compared the iron status of women following omnivore and vegetarian diets. Most have found vegetarians to have lower serum ferritin concentrations than omnivores [57,68,69,70]. Premenopausal women in the United States who consumed red meat had higher serum ferritin and hemoglobin concentrations, and lower total iron binding capacity compared with lacto-ovo vegetarians, or those consuming fish or poultry as their main protein source [64]. However, in the United Kingdom, serum ferritin concentrations (and total iron intake) were lower in women who ate red meat at least five times per week compared with those who avoided red meat but consumed fish and poultry at least five times per week [51]. Consumption of a vegetarian diet was not associated with reduced serum ferritin concentrations, however, transferrin receptor concentrations were significantly higher in women who consumed a vegetarian diet compared with women who habitually ate red meat, or fish and poultry, suggesting a higher degree of iron deficiency in vegetarians [51]. Several studies have found a similar incidence of iron deficiency anemia in vegetarians and vegans compared with omnivores [68,69,71].

### 7.3. Fruit and Vegetables/Ascorbic Acid

Although ascorbic acid is recognized as a powerful enhancer of iron absorption, the majority of studies in young women have not found any association between iron status and total daily ascorbic acid intake [45,46,47,48,49,50,53,56,57,60,61,67,72,73], or fruit and vegetable intake [50,54,59,60,74]. A study in females aged 35 to 69 years found a negative association between intake of fruit juice and iron status, but a positive association between ascorbic acid intake and iron status [54].

### 7.4. Vitamin A/β-Carotene

Despite the emergence of a potential role for β-Carotene and vitamin A in iron absorption [75], only one cross-sectional study has considered vitamin A intake and found no association with iron status [50].

### 7.5. Dairy Products/Calcium

Several studies in young women have found a negative association between iron status and calcium [53,60] or dairy product [47,60] intake. Across Europe, van de Vijver* et al*. [76] found dietary calcium intake to be inversely associated with serum ferritin concentration, regardless of whether calcium was consumed with iron. Other studies, however, have found no association between iron status and calcium [47,48,49,50,54,56,57] or dairy product or milk intake [45,49,50,59,66]. It has been suggested that in some cases, the negative associations between iron status and calcium or dairy product intake may be due to the displacement of other foods from the diet (e.g., meat), rather than the inhibitory effect of calcium itself [48].

### 7.6. Bread and Cereals/Fibre/Phytate

In a group of middle-aged females (35 to 69 years), Cade* et al.* [54] found white and wholemeal bread, and nut and seed consumption were negatively associated with serum ferritin concentrations. As far as we are aware, only one study in young women has found a negative association between fibre intake and serum ferritin concentration [60], with most studies not reporting any association between phytate [48,50,67] or fibre [45,50,54,56,57,61] intake and iron status. Other studies have found no association between iron status and cereal and pulse intake in young Japanese women [50], cereals and legumes in Spanish women or between iron status and consumption of beans and pulses, or fortified breakfast cereals in women aged 35 to 69 years living in the UK [54]. In Australia, Leonard et al found no association between iron status (serum ferritin, hemoglobin or soluble transferrin: serum ferritin index) and intake of baked beans, soy beans, other beans, iron fortified breakfast cereals, rice, or pasta [57].

Peneau* et al*. [74] found no association between serum ferritin concentration and consumption of ascorbic acid-rich fruit, vegetables and juices. However higher intakes of fibre-poor fruit and vegetables juices were associated with higher serum ferritin concentrations. The authors suggested that in diets containing a range of fruit and vegetable combinations, the effects of differing levels of ascorbic acid and fibre may counteract one other, leading to no change in serum ferritin concentration [74].

### 7.7. Protein Based Foods/Soy Protein

Most studies have not reported on associations between iron status and protein intake. Brussard *et al*., observed a negative association between vegetable protein intakes and iron status [45], and Blanco-Rojo *et al*., found no association between protein intake and women with normal iron status, mild iron deficiency and women close to developing iron deficiency anaemia [59]. Leonard* et al*. [57] observed serum ferritin concentrations to be negatively associated with frequency of egg intake, however, other studies found no association between iron status and egg consumption [45,50,59,66].

### 7.8. Tea and Coffee/Polyphenols

The majority of studies have found no relationship between tea and coffee intake [45,48,60,66,77] or polyphenol intake and iron status [54,67]. Three studies have found a negative relationship between serum ferritin concentration and tea intake [46,47,49]. A review by Temme and van Hoydonck (2002) concluded tea consumption does not appear to affect iron status in populations who mostly have adequate iron status. However, in populations with marginal iron status, there appears to be a negative association between tea consumption and iron status [78].

### 7.9. Alcohol

Studies investigating alcohol intake have found either a positive [45,49,54,63,79,80] or no association [50,53] with iron status. The type of alcohol may be important, with beer rather than wine or spirits being associated with a higher serum ferritin concentration in two studies [63,79]. In pre-menopausal Finnish blood donors, frequency of wine intake was correlated with higher serum ferritin concentrations, however, beer and liquor had no effect. Both wine and beer intake were associated with a reduced risk of iron deficiency (SF < 15 µg/L) [66]. Using data from the third National Health and Examination Survey in the United States, Ioannou* et al*. [81] concluded that consumption of up to two alcoholic drinks per day was associated with reduced risk of iron deficiency and iron deficiency anemia in adult men and women.

### 7.10. Food and Nutrients Consumed in Combination

The combinations of foods and beverages eaten at meal times or across the day may be a more important indicator of iron status than the investigation of individual foods and nutrients, as foods are most likely to impact on non-heme iron absorption when consumed simultaneously [73,82,83].

#### 7.10.1. Timing of Food Consumption and Iron Status

Only a few studies have investigated associations between iron status and the timing of foods consumption [46,76,77] or different combinations of foods eaten at mealtimes [84]. In a small study (*n* = 15) of women with learning difficulties who lived in an institutionalized setting, those who had depleted iron stores (SF < 12 µg/L) had a significantly higher tea and lower ascorbic acid intake at meal times [46] compared to women who had higher iron stores (SF > 12 µg/L). These women’s diets were likely to be more prescribed than those of women living independently, where a range of foods are consumed in a variety of combinations across the day [84]. Mennen* et al*. [77] found tea drinking with or between meals was not associated with serum ferritin concentrations or iron depletion in French adults. In another study, the timing of calcium intake (*i.e*., consumed with iron or not) had no effect on iron status [76]. Using a non-validated questionnaire, Beck* et al*. [84] found women with sufficient iron stores (SF ≥ 20 µg/L) were more likely to consume milk or milk products between meals compared with women who had suboptimal iron status (SF < 20 µg/L). The consumption of other foods and beverages in various combinations at meals and in between meals was not associated with iron status. Despite including 375 women, the sample size may not have been large enough to detect associations between iron status and all possible combinations of foods and beverages consumed [84]. Further research is needed to investigate the relationship between timing of food and beverage intake and iron status.

#### 7.10.2. Dietary Patterns and Iron Status

Another approach to assessing associations between combinations of foods and beverages consumed and iron status is to focus on dietary patterns. Dietary patterns consider the whole diet and how foods are consumed in combination [85,86], rather than the traditional approach which investigates individual foods and nutrients [85,86]. This approach is particularly useful in iron-related nutrition, where multiple foods and nutrients impact on iron absorption. Two main approaches can be used to determine dietary patterns—a theoretical approach, whereby foods and nutrients are grouped according a set criteria for nutritional health (e.g., a dietary index which ranks dietary intake according to the iron bioavailability of a diet), or an empirical approach, where foods and nutrients are reduced into a smaller number of variables [85,86], typically based upon inter-correlations between dietary items (factor analysis), or individual differences in mean intake (cluster analysis) [85,86].

As far as we are aware, only three studies have explored the association between dietary patterns and iron status [84,87,88], and only one has focused on young women [84]. Using a validated iron food frequency questionnaire [89], Beck* et al*. [84] observed a decreased odds of suboptimal iron status (SF < 20 μg/L) in women aged 18–44 years who followed a “meat and vegetable” dietary pattern, and an increased odds of suboptimal iron status in women who followed a “milk and yoghurt” dietary pattern. No associations were observed between iron status and the following dietary patterns: refined carbohydrate and fat, Asian, healthy snacks, high tea and coffee and bread and crackers [84]. However, the impact of dietary patterns on iron status was reduced when other non-dietary determinants of iron status were considered [90].

### 7.11. Limitations of Studies Investigating Associations between Dietary Intake and Iron Status

The majority of studies investigating associations between dietary intake and iron status have been based on cross-sectional study designs. Cross-sectional studies are unable to identify causal relationships, as exposure (diet) and outcome (iron status) are measured at the same time [91]. It is also difficult to compare the findings from cross-sectional studies due to differences in the populations studied (e.g., age, country), differences in biochemical indices and cut-offs used to define iron deficiency, the analysis of results which does not account for non-dietary factors than impact on iron status (e.g., blood loss, genetics) and varying methods of assessing dietary intake [20]. There are also limitations associated with the various dietary assessment methods that have been used [92]. For example, it is recommended that 11 days of dietary record data are needed to accurately assess iron intake when food records are used [93], and this is usually not achieved. There may also be limitations with food composition data used. For example, New Zealand does not include phytic acid or polyphenols in their national food composition database. Intervention studies provide a more robust study design for investigating the effect of dietary intake. These are discussed in Section 8.

### 7.12. Conclusions—Cross-Sectional Studies

In summary, most cross-sectional studies in young women living in industrialized countries have found meat or heme iron consumption to be associated with an increased iron status. Some, but not all studies, have observed a negative association between calcium and dairy product intake and iron status. Studies have either found a positive association or no association between alcohol intake and iron status. Tea intake appears to only impact on iron status in women with marginal iron status. The majority of cross-sectional studies in young women living in industrialized countries have observed no relationship between iron status and intakes of total iron, ascorbic acid, fruit and vegetables, fibre, and phytate. Recent studies have investigated the relationship between iron status and combinations of food and beverages eaten with mixed results. The limitations of cross-sectional studies should be considered when interpreting their findings.

## 8. Dietary Solutions to Iron Deficiency for Young Women Living in Industrialized Countries

Iron deficiency can be treated through a range of measures including dietary interventions (including education) to increase the intake and bioavailability of iron in the diet [26], iron fortification of food or through iron supplementation. This section of the review focuses on dietary interventions aimed at improving iron status in young women.

### 8.1. Interventions Using Meat

Four studies in young women have investigated the effect of meat intake on iron status [94,95,96,97] (Table 4). Lyle* et al*. [94] compared the effects of increasing meat intake and oral iron supplementation in a group of previously sedentary women living in university halls of residence who took part in a 12 week moderate exercise program. Between baseline and 12 weeks, serum ferritin concentrations decreased significantly in women who consumed a free choice diet and placebo (8.8 mg total iron; 1.0 mg heme iron/day). Serum ferritin concentrations also decreased significantly in a non-exercising control group who consumed a diet of free choice (8.0 mg total iron; 0.8 mg heme iron/day). Serum ferritin concentrations decreased (but not significantly) in women who consumed a 10 mg elemental iron supplement (17.5 mg total iron; 0.4 mg heme iron/day), and did not change in women who consumed a 50 mg elemental iron supplement each day (57.8 mg total iron; 0.6 mg heme iron/day). For women who increased meat intake (11.8 mg total iron; 1.8 mg heme iron/day), serum ferritin concentrations increased, but not significantly [94].

An early cross-over study in which all food was provided found serum ferritin or hemoglobin concentrations did not improve in women who consumed either a omnivore diet (184 g meat; 17.3 mg total iron; 1.5 mg heme iron/day) or a lacto-ovo vegetarian (0 g meat; 17.8 mg total iron/day) for eight weeks [95]. This study was limited by a short duration of intervention, and included participants who had high serum ferritin concentrations (less likely to absorb iron). A more recent cross-over study also of short duration, but which included iron-deficient women (SF < 30 µg/L, Hb > 110 g/L), found no significant differences in serum ferritin, hemoglobin or soluble transferrin receptor concentrations of women who consumed five of eight portions of meat per week as oily fish (11.5 mg iron/day) compared with five of eight portions of meat as red meat (13.9 mg iron/day) for eight weeks [96].

In a well-designed 20 week intervention study, Tetens* et al.* [97] found women with low iron stores (SF ≤ 30 µg/L, Hb > 120 g/L) who consumed 150 g meat per day (pork, beef or chicken) (approximately 2.4 mg iron) in addition to their usual diet had no significant changes in serum ferritin or hemoglobin concentrations [97]. However, women who consumed vegetable products of similar energy and iron content (to the meat products) and consumed less than 250 g meat per week had a significant decrease in serum ferritin and hemoglobin concentrations [97]. The results of these studies are shown in Table 4.

### 8.2. Interventions Using Dietary Ascorbic Acid

Several studies have found no effect on iron status when participants consume ascorbic acid supplements with meals [43,98,99]. However, these studies have had a number of limitations, including no control groups [98], small sample sizes [43,98,99], short duration of intervention [43,99], and the inclusion of iron replete participants who are less likely to absorb iron [98]. Both synthetic and naturally occurring ascorbic acid in foods increase iron absorption [73,100,101]. This review focuses on interventions using foods rich in ascorbic acid to improve iron status in young women (see Table 5).

Using a 30 day cross-over study design, Kandiah [102] found an increase in hemoglobin concentrations but no changes in serum ferritin concentrations in 14 young vegetarian women who consumed their normal diet with tofu and orange juice (101 mg ascorbic acid), compared with normal diet and tofu alone at mealtimes. This study had several limitations including a short duration of intervention and a small sample size. Iron status was not reported at baseline for all of the women involved. A number of well-designed intervention studies have provided further insight into the effect of ascorbic acid on iron status in young women [103,104,105].

In an eight-month intervention study, Garcia *et al*. [104] found that lime juice (25 mg ascorbic acid) compared with a lime-flavored beverage (0 mg ascorbic acid) consumed with two meals per day for six days each week did not improve serum ferritin or hemoglobin concentrations in 18 iron deficient (SF < 12 μg/L) women [102]. This was despite a previous study by the same research group showing that the lime juice more than doubled iron absorption in iron deficient women (SF < 12 μg/L) [100]. Both the intervention and control group consumed similar amounts of iron, and both groups had diets high in non-heme iron and phytate. The authors suggested that high menstrual blood losses meant improvements in iron absorption [100] were insufficient to increase iron stores [104], and that other micronutrient deficiencies (e.g., vitamin A) may have confounded any enhancing effect on iron status [104]. This study did not report the amount of iron consumed alongside the lime juice. Due to ascorbic acid’s mechanistic action, ascorbic acid is more likely to improve iron status if consumed with substantial amounts of fortificant iron [104]. Two studies, both of 16 weeks in duration, have since added ascorbic acid rich foods to meals containing large amounts of iron in women with non-anemic iron deficiency [103,105].

Using a 16 week randomized controlled intervention trial in 69 young women with low iron stores (SF < 25 µg/L, Hb ≥ 115 g/L), Beck* et al*. [103] found that the daily addition of two gold kiwifruit (164 mg ascorbic acid) compared with a banana (ascorbic acid not detected) to an iron fortified breakfast cereal (16 mg iron as ferrous sulphate) and milk meal improved iron status. Serum ferritin and soluble transferrin receptor concentrations improved significantly in the kiwifruit group but not the banana group, and the change between groups was significant. Hemoglobin concentrations increased significantly in the kiwifruit group, but not the banana group; the change between groups was not statistically significant. The only dietary change required of participants was the addition or replacement of their usual breakfast meal with a standardized breakfast meal. Women were asked not to consume any other food or fluid one hour either side of this meal. Participants were counseled at baseline regarding these dietary changes and followed up regularly (six times either by phone or email) throughout the intervention in order to maintain compliance.

**Table 4:** Interventions using meat to improve to improve iron status in young women.

Author, Country	Participants, Duration of Intervention	Dietary Intervention	Serum Ferritin (µg/L) Baseline/End	Hemoglobin (g/L) Baseline/End	Final Outcome
Navas-Carreterro* et al.* [96] Spain	25 females, 18–30 y, SF < 30 µg/L, Hb > 110 g/L 8-week random cross-over	Red meat diet—usual diet and 5 portions red meat, 1 portion lean fish, 2 portions poultry, 2 eggs/week Oily fish diet—usual diet and 2 portions salmon, 1 portion tuna, 1 portion sardines, 1 portion lean fish, 1 portion red meat, 2 portions poultry, 2 eggs/week	n/a n/a	n/a n/a	No significant differences in SF, Hb or sTfR between groups
Tetens* et al.* [97] Sweden	57 females, 19–39 y SF ≤ 30 µg/L, Hb > 120 g/L 30-week RCT	Meat based diet—usual diet and 150 g meat/day (pork, beef or chicken) Vegetable based diet—usual diet and maximum 260 g meat and 125 g fish/week	16.3/16.5 (NS) 17.3/11.2 (*p* < 0.001)	126.0/125.0 (NS) 124.0/121.0 (*p* = 0.003)	Significant difference in SF and Hb between groups
Hunt and Roughead [95] USA	21 females, 20–42 y, SF 6–149 µg/L 8 week random cross-over	Lacto-ovo-vegetarian diet (0 g meat/day) Omnivore diet (184 g meat/day)	22.0 ^a^ 22.0 ^a^	133.0 ^a^ 135.0 ^a^	No significant effect of diet on SF or Hb
Lyle* et al*. [94] USA	60 females, 18–19 y (mean) 12-week RCT	50 mg iron supplement as ferrous sulphate, low iron diet, exercise (57.8 mg iron/day) 10 mg iron supplement as ferrous sulphate, low iron diet and exercise (17.5 mg iron/day) Placebo, free choice diet and exercise (8.8 mg iron/day) High iron diet (meat supplement) and exercise (11.8 mg iron/day) Control- free-choice diet and no exercise (8.0 mg iron/day)	27.0/27.5 (NS) 48.9/34.7 (NS) 40.0/23.9 (*p* < 0.05) 23.7/29.2 (NS) 22.2/12.7 (*p* < 0.05)	126.0/124.0 (NS) 129.0/125.0 (*p* < 0.05) 120.0/115.0 (*p* < 0.05) 116.0/124.0 (*p* < 0.05) 121.0/121.0 (NS)	SF of 50 mg ferrous sulphate and high iron diet groups significantly higher than control group, and high iron diet significantly higher than placebo group at 12 weeks Hb of high iron diet significantly higher than other groups at 12 weeks

Table adapted from “Iron and Health” Scientific Advisory Committee on Nutrition [20]; ^a^: Mean serum ferritin and hemoglobin following 8-weeks consumption of vegetarian and omnivore diet. Abbreviations: Hb—Hemoglobin; n/a—not available; NS—non significant; RCT—Randomized controlled trial; SF—Serum ferritin; sTfR—Soluble transferrin receptor.

**Table 5:** Interventions using dietary ascorbic acid to improve to improve iron status in young women [20].

Author, Country	Participants, Duration of Intervention	Dietary Intervention	Serum Ferritin (µg/L) Baseline/End	Hemoglobin (g/L) Baseline/End	Final Outcome
Beck* et al*. [103] New Zealand	69 females, 18–44 y, SF ≤ 25 µg/L, Hb ≥ 115 g/L 16-week RCT	Intervention—normal diet and iron-fortified breakfast cereal (16 mg iron as ferrous sulphate), milk, 2 gold kiwifruit (164 mg Aa) Intervention-normal diet and iron-fortified breakfast cereal (16 mg iron as ferrous sulphate), milk, 1 banana (0 mg Aa)	17.0/25.0 (*p* < 0.001) 16.5/17.5 (NS)	126.0/130.0 (*p* = 0.005) 125.0/126.0 (*p* = 0.30)	Significant increase in SF and decrease in sTfR in kiwifruit but not banana group, change between groups significant Significant increase in Hb in kiwifruit but not banana group, no significant change between groups
Garcia-Casal *et al*. [104] Mexico	36 females, 28 y (mean), SF < 12 µg/L 8-mth RPC	Intervention—normal diet and limeade (25 mg Aa) at 2 meals, 6 days/week Placebo—normal diet and limeade (no Aa) at 2 meals, 6 days/week	6.4/9.0 (NS) 6.2/8.7 (NS)	137.0/140.0 (NS) 139.0/137.0 (NS)	No significant differences between groups in SF, Hb, sTfR, sTfR:SF ratio
Kandiah [102] USA	14 females, 20–25 y, vegetarian, 1/3 had SF < 12 µg/L 30-d random cross-over	Intervention—normal diet and 57.7 g tofu & 83.3 mL (101 mg Aa) orange juice 3x/day with meals Placebo—normal diet and 57.7 g tofu 3x/day with meals	n/a n/a	8.1 & 6.4 ^a^ −5.5 and −6.5 ^a^	No significant difference in SF between groups Hb increased significantly with orange juice compared with no orange juice

Table adapted from “Iron and Health” Scientific Advisory Committee on Nutrition [20];^ a^: Change in Hb concentrations during first and second experimental period. Abbreviations: Aa—Ascorbic acid; Hb—Hemoglobin; n/a—not available; NS—non significant; RCT—Randomized controlled trial; RPC—Randomized placebo controlled; SF—Serum ferritin; sTfR—Soluble transferrin receptor.

A recent study provides support to the theory that ascorbic acid should be consumed with substantial amounts of iron in order to improve iron status. In a randomized double blind placebo controlled trial in 122 iron deficient women (SF ≤ 40 µg/L, Hb ≥ 110 g/L), serum ferritin, soluble transferrin receptor and hemoglobin concentrations improved in women who consumed an iron-fortified fruit juice (containing 18 mg iron as microencapsulated iron pyrophosphate and 95 mg ascorbic acid) in addition to their normal diet after 16 weeks. Iron status did not improve in women who consumed the unfortified fruit juice only (*i.e*., ascorbic acid, but no iron) [105].

### 8.3. Interventions Using a Combination of Dietary Measures

Surprisingly, only two intervention studies have used a combination of dietary approaches to improve iron status in young women [106,107]. In the first study, a 16-week dietary intervention improved serum ferritin concentrations but not significantly compared with a placebo group, while the addition of a daily iron supplement (50 mg elemental iron) significantly improved serum ferritin concentrations in women with mild iron deficiency (SF < 20 µg/L, Hb ≥ 120 g/L) [106]. Soluble transferrin receptor showed no change in the dietary group, but decreased in the supplement group, and hemoglobin concentrations did not change significantly in any group. The dietary intervention is detailed in Table 6, and included advice to: increase the intake of iron-containing foods and enhancers of non-heme iron absorption, decrease the intake of iron absorption inhibitors, and to consume enhancers of non-heme iron absorption at meal times, and inhibitors between meals. Participants received individualized dietary advice, and face-to-face and telephone counseling throughout the intervention. They were provided with 250 mL fruit juice per day, a vitamin C rich fruit syrup, recipe books and a cast-iron frying pan. Women in the dietary intervention group significantly increased their intake of meat, fish and poultry (31 g/day compared with the placebo group), heme iron (0.36 mg/day), ascorbic acid (136 mg/day) and foods cooked using cast-iron cook ware; and significantly decreased their calcium intake (169 mg/day) and their phytate: iron molar ratio (5.6 compared with 8.5 in the placebo group), but there was no significant change in total iron intake (range 11.0–12.4 mg/day) across groups during the intervention [106].

In a 12-week intervention, Patterson* et al*. [107] found a highly iron-bioavailable diet (aimed at providing 2.25 mg absorbable iron/day) produced smaller but significant increases in serum ferritin compared with daily iron supplementation (105 mg elemental iron) in women with low iron stores (SF < 20 µg/L, Hb > 90 g/L). Hemoglobin concentrations increased in both groups, but only significantly in the supplement group. Participants were given dietary advice regarding combinations of high, medium and low iron foods to consume each day, were encouraged to consume enhancers of iron absorption at each meal, and to avoid tea, coffee or milk at lunch and dinner. Participants received counseling from a dietitian, and were provided with meat vouchers to purchase 120 g beef or lamb per day. Women in the diet group did not significantly change their intake of heme iron, non-heme iron, ascorbic acid or phytate. Total iron intake at the end of the intervention ranged from 10.7 to 12.8 mg iron/day across each group. This suggests the combinations of foods consumed may have been more important than the total amount of each nutrient consumed on a daily basis. A control group with replete iron stores (SF > 20 µg/L, Hb > 120 g/L) showed no significant change in their iron status over the course of the intervention. After six months, serum ferritin concentrations continued to increase in the diet group, but remained stable in the supplement group [107]. While both of these studies have shown improvements in serum ferritin concentrations, it has not been enough to move women from a depleted to an iron replete state [106,107].

### 8.4. The Effect of Consuming Dietary Inhibitors of Iron Absorption on Iron Status

It is also important to consider the effects of inhibitors of iron absorption on iron status, as these inhibitors may counteract interventions aiming to improve iron status in young women. Studies in young women have found no effect of calcium supplementation on serum ferritin or hemoglobin concentrations in young women [108,109]. A review by Bendich [110] concluded that calcium supplementation as high as 1200 mg per day does not affect iron status in healthy premenopausal women. Further studies are needed to determine whether calcium intake affects iron status in women who are anemic or have low iron stores [110]. Intervention studies have not yet investigated the effect of calcium from dietary sources on iron status. However, calcium in food has been shown to inhibit iron absorption to a similar extent to calcium obtained from supplements [111].

Only one intervention study has investigated the effects of dietary fibre intake on iron status over the longer term. Bach-Kristensen* et al*. [112] found consumption of a high fibre bread (providing the recommended dietary intake for fibre) as a substitute for part of the normal diet (breakfast cereals, bread for lunch and afternoon snacks) over a four-month period reduced serum ferritin concentrations significantly (from 44 to 34 µg/L) in women with replete iron stores (SF > 20 µg/L, Hb > 120 g/L). Another group of women consumed the bread with added phytase (reducing the bread’s phytic acid: iron molar ratio from 8.5:1 to 6.7:1). The reduction in phytic acid content was not adequate to maintain serum ferritin concentrations with levels falling from 45 to 32 µg/L.

Intervention studies investigating the effect of adding protein or phytate (in the form of soy protein) to the diet have shown mixed results [113,114,115], possibly due in part to variable phytic acid: iron molar ratios. Swain* et al*. [113] found serum ferritin concentrations increased when women consumed either 40 g of soy protein isolate or whey protein per day as a meal replacement over a 24 week period. No negative effects on iron status were observed in women who consumed beef products extended with varying amounts and types of soy protein over a six month study period [114]. A recent study in the United States found adding soy-based foods (19 g soy protein/day) to the diet over 10 weeks had no effect on iron status in premenopausal women [115]. No intervention studies in young women have investigated the effect of other non-meat based proteins, or tea and coffee intake on iron status over the longer term.

**Table 6:** Interventions using combined dietary approaches to improve iron status in young women.

Author, Date, Country	Participants, Duration of Intervention	Dietary Intervention	Serum Ferritin (µg/L) Baseline/End	Hemoglobin (g/L) Baseline/End	Final Outcome
Heath* et al*. [106] New Zealand	75 females, 18–40 y SF < 20 µg/L, Hb > 120 g/L 16-week RPC	Placebo Iron supplement (50 mg iron/day as amino acid chelate) Diet (advice to: increase intake of iron containing foods, foods containing factors that enhance iron absorption; decrease intake of foods containing factors that inhibit iron absorption, modify eating patterns so enhancers of iron absorption are eaten with meals and potential inhibitors in between meals	11.7/12.6 (NS) 8.4/16.8 (*p* = 0.001) 10.3/14.0 (*p* = 0.068)	131.6/133.0 (NS) 130.9/133.4 (NS) 131.6/132.2 (NS)	SF increased in the supplement (significantly) and diet groups (NS) sTfR decreased in supplement group, but no change in diet group NS changes in Hb for any group
Patterson* et al*. [107] Australia	44 females, ≥18 y SF < 15 or SF 15–20 µg/L and serum iron <10 µmol/L and TIBC > 68 µmol/L 12-week RCT	Iron supplement (105 mg iron/day as ferrous sulphate) Diet (planned to provide 2.25 mg absorbable iron using combinations of high/medium/low iron foods; encouraged subjects to consume meat and vitamin C rich foods at each meal; consumption of tea, coffee, milk were discouraged at lunch and dinner) Control group (SF > 20 µg/L, Hb > 120g/L)	9.0/24.8 (*p* < 0.05) 8.9/11.0 (*p* < 0.05) 49.4/44.5 (NS)	125.2/130.4 (*p* < 0.05) 127.6/130.6 (NS) 135.9/134.0 (NS)	Significant increase in SF in diet and supplement groups Significant increase in Hb in supplement group

Abbreviations: Hb—Hemoglobin; NS—non significant; RCT—Randomized controlled trial; RPC—Randomized placebo controlled; SF—Serum ferritin; sTfR—Soluble transferrin receptor; TIBC—Total iron binding capacity.

### 8.5. Limitations of Studies Investigating the Effect of Dietary Interventions on Iron Status

Intervention studies provide a better insight than cross-sectional studies into to the effects of dietary intake on iron status. These studies have, however, been limited by small sample sizes and, therefore, possibly insufficient power to show an effect [95,96,102], and the use of participants with normal rather than low iron stores [94,95,102]. A measurable effect of dietary interventions on iron status may only be seen in populations with a low iron status, who absorb more iron from the diet [116]. In addition, it is possible that when participants have low hemoglobin concentrations, absorbed iron is preferentially used for hemoglobin synthesis before serum ferritin concentrations (iron stores) are increased, and some studies have not been undertaken for long enough for this to be observed [95,96,102] Interventions should be at least 12–16 weeks duration to allow adequate time for red blood cell turnover (red blood cells have a life-span of 90–120 days) [117]. The baseline diet to which food is added will impact on how much iron is absorbed. Less iron will be absorbed when foods or nutrients are added to a diet with high iron bioavailability compared with adding foods or nutrients to a diet of lower bioavailability [73]. Some studies have only reported total dietary iron intake, and not the iron content of the meal to which the dietary intervention was added [104]. Furthermore, not all dietary intervention studies have controlled for other factors that may affect iron status including blood loss and supplement use.

### 8.6. Conclusions—Intervention Studies

Intervention studies investigating the effect of meat and ascorbic acid on iron status have shown mixed results. In longer term studies (>12 weeks), meat consumption appears to maintain or improve iron status. Ascorbic acid appears to increase iron status in iron deficient women only when consumed together with substantial amounts of iron. A combination of approaches (e.g., increased meat and ascorbic acid intake, decreased intake of iron absorption inhibitors and considered timing of enhancers and inhibitors of iron absorption) appears to offer the most effective dietary means of addressing iron deficiency in young women living in industrialized countries.

## 9. Conclusions

Young women living in industrialized countries are vulnerable to iron deficiency even though the typical diet in these countries tends to have higher bioavailability because it has more heme iron and fewer inhibitors of iron absorption. Although many studies have demonstrated effects of manipulating dietary components which inhibit or enhance iron absorption in single meal studies, there is little association between total iron intake and iron status over a longer period. This may be because the balance between dietary factors enhancing iron uptake and those inhibiting it is not adequately considered. Recent studies have considered how foods and beverages are eaten in combination, through the use of dietary pattern analysis. The most successful dietary approaches for treating iron deficiency appear to be those that use multiple approaches to increase iron status. For example, increased intake of iron (particularly heme iron) and enhancers of iron absorption; decreased intake of iron absorption inhibitors, as well as optimal timing of enhancers and inhibitors of iron absorption. More longitudinal studies and randomized controlled trials are needed to investigate associations and the effects of dietary interventions on iron status in young women living in industrialized countries. Dietary interventions for treating iron deficiency must consider the intervention meal, including the iron content and other enhancers and inhibitors of iron absorption that may be consumed with the meal, as well as the possible displacement of foods from the normal baseline diet. These interventions should include women who have low iron stores, but are not anemic, and should be of at least 12–16 weeks in duration in order to allow time for an effect to be seen. Power calculations should be undertaken to ensure sample sizes are adequate to detect clinically significant differences in biochemical iron indicators. Interventions should control for confounders such as blood loss and supplement use.
